# The regulatory cliff edge between contraception and abortion: the legal and moral significance of implantation

**DOI:** 10.1136/medethics-2015-102712

**Published:** 2015-06-17

**Authors:** Sally Sheldon

**Keywords:** Abortion, Bills, Laws and Cases, Law, Legal Aspects, Reproductive Medicine

## Abstract

In regulating the voluntary interruption of pregnancy, English law has accorded particular significance to two biological events. First, ‘viability’, the moment when a fetus is said to acquire the capacity for independent life, plays an important role in grounding restrictions on access to legal abortion later in pregnancy. Second, equally significantly but far less frequently discussed, ‘implantation’ marks the point in pregnancy from which abortion laws apply. This paper focuses on this earlier biological event. It suggests that an unquestioning reliance on implantation as marking an appropriate moment of transition between two radically different legal frameworks is deeply problematic and is rendered still less sustainable in the light of the development of new technologies that potentially operate shortly after the moment of implantation.

## Introduction

Scientists have suggested that the development of ‘contragestive’ technologies (which operate in the early weeks after intercourse, at the borderline between contraception and abortion) is technically possible and might offer significant new means of fertility control, which have clear advantages for some women.^1^ However, it would be difficult, if not impossible, to make these new technologies available within England. This is not as a result of modern, democratic deliberation of the ethical and clinical merits and risks of such treatments but is rather an unconsidered consequence of archaic legislation, framed at a time when they were unimaginable. This legislation offers a ‘regulatory cliff edge’ between methods of fertility control that operate before implantation and those that operate after it. In this paper, I argue that this status quo is morally indefensible. I begin with a brief discussion of biology and the relevant law, before moving on to assess the implications of new technologies and the moral significance of implantation. While I focus on English law, the broad issues raised are also relevant to many other countries that take implantation as marking a legal boundary between contraception and abortion.

## Seamless biology and legal bright lines

As Glanville Williams once said ‘[a]bstract human life does not ‘begin’; it just keeps going’.^2^ A seamless biological continuum exists through the production of sperm and egg, their joining together in a process of fertilisation, the gradual development of the new entity thus created throughout pregnancy, birth, subsequent growth, eventual death and ensuing decay of the body. Defining what happens along the way as an ‘embryo’, ‘fetus’, ‘person’, ‘adult’, or ‘corpse’ requires an attempt to draw bright lines on the basis of criteria selected as holding significance for legal or other purposes. The drawing of such lines is challenging and not only because the selection of the relevant criteria will inevitably be contested. Also, the issue of when each of these various statuses is achieved is complicated because any characteristics on which they rely are liable to develop at a different number of hours, days, weeks or years in different organisms. Further, the characteristics may appear or be lost gradually over time. Implantation, for example, involves the fertilised egg physically attaching itself to the wall of the womb some 6–12 days after ovulation.^3^ It occurs as a process that occurs in a number of distinct stages, including apposition (where the blastocyst orients itself correctly), the shedding of the zona pellucida, adhesion (an attachment to the endometrial surface) and invasion and embedding (its further establishment).^4^

Implantation is, in many countries, taken to represent the dividing line between abortion and contraception, with these two practices generally treated as if they are readily distinguishable and appropriately subject to very different regulation. While the need for some elements of prescription-only status and pharmacist control has at times been contested, the regulation of contraceptives in Britain primarily reflects health considerations, imposing varying levels of control that reflect the risks of side effects and the technical complexity of the method used.^5^ ^6^ Thus, condoms are freely available for purchase in shops, the combined pill is available on prescription only and the morning-after pill is available over the counter from a pharmacist. This framework stands in stark contrast to the morally grounded criminal prohibitions that establish the parameters within which abortion services may be legally offered. While also said to be informed by a concern for women's health, it is generally accepted that this latter framework aims to limit or to condemn the intentional destruction of fetal life.^7^ ^8^ Thus, the Offences Against the Person Act 1861 provides for a potential sentence of life imprisonment for any unlawful ‘procurement of miscarriage’, from the moment that a pregnancy is established through implantation, and a 5 year prison sentence for the unlawful supply or procurement of any poison, other ‘noxious thing’ or instrument for that purpose.^9^ Intentional disruption of a pregnancy will thenceforth be lawful only where offered in line with the therapeutic exception carved out by the Abortion Act 1967 which, in all but emergency circumstances, requires that a termination is authorised by two doctors and performed by a doctor on approved premises.^10^

With the key phrase of ‘procurement of miscarriage’ left undefined in the 1861 Act, it has fallen to the courts to clarify its meaning and, thus, to decide from which moment in the biological process the Act should apply. In a lengthy and erudite judgement in the case of *Smeaton*, Munby J (as he then was) confirmed that the morning-after pill, which operates post fertilisation but prior to implantation, was a contraceptive rather than an abortifacient.^i^ The judge reasoned that the offence of ‘unlawful procurement of miscarriage’ presupposes some prior ‘carriage’ that involves ‘not merely presence in the woman's body and interaction with it, but attachment to it in a real sense such as occurs only with implantation’ (para 353).^11^ This ruling, reflecting modern medical understandings of the term ‘miscarriage’, allowed the Court to avoid the ‘unattractive’ possibility that ‘a judge in 2002 were to be compelled by a statute 141 years old to hold that what thousands, hundreds of thousands, indeed millions, of ordinary honest, decent, law abiding citizens have been doing day in day out for so many years is and always has been criminal’ (para 394).^11^ While achieving a practical, workable outcome for the morning-after pill, however, drawing a bright line at the moment of implantation offers a far from satisfactory basis for the future development of techniques of fertility control that operate in early pregnancy. Further, the regulatory ‘cliff edge’ between abortion and contraception is likely to gain greater significance in the future, raising issues that can only be resolved by statutory reform.

## Implantation and contragestive technologies

Researchers are currently exploring the potential to develop new ‘contragestive’ drugs that operate around the time of implantation, including some treatments that might operate either before or after it has occurred. The legal issues raised by such potential regimens are not entirely new. In the 1970s, a doctor reported that he offered menstrual regulation within 10–18 days of a missed period, without first confirming whether a woman was pregnant.^12^ Where offered post implantation, it was accepted that this practice was caught by the criminal prohibition contained in the 1861 Act, with opinion divided regarding whether it might be rendered lawful even where attempts were made to comply with the Abortion Act: in particular, given that this latter statute applies only when a pregnancy is ‘terminated’, can it render lawful a procedure carried out where a pregnancy has not been diagnosed?^13^ The answer remains uncertain in the absence of a court decision.^13^

New drug-based regimens that would operate very early in pregnancy raise similar issues. For example, researchers have raised the possibility of developing treatments that a woman might potentially use on a planned schedule only once in each menstrual cycle, no matter how many prior coital acts she had had over that period.^1^ ^14^ Such drugs might potentially act either before or after implantation. A further possibility might be to limit the use of drugs to, on average, a few times a year, when a woman's menstrual period was late.^1^ Such drugs would be effective for a longer period following intercourse than emergency contraceptives that work only if taken before ovulation, and therefore they could potentially serve more women and provide more benefit at the population level.^1^ ^15^

While such regimens would not be chosen by all women, they would extend the range of current fertility control methods in ways that might be attractive and beneficial for some. First, postfertilisation methods eliminate the conceptual and logistical challenge of needing to obtain and initiate contraception before having sex, which can be daunting.^1^ Second, the latter regime (where a woman would wait for a missed period before taking the drug) would mean that she would be less likely to take medication unnecessarily: while the reason for taking the morning-after pill is the actual fact of unprotected sex, here, a missed period means a greater likelihood that a pregnancy exists.^1^ Third, the development of such techniques might also offer considerable advantages in terms of convenience for women, if it were shown to be safe for the drugs to be obtained in advance and kept in the bathroom cabinet to be used if necessary. Such ready access to early treatment might potentially decrease the need for later abortions. Fourth, the regimens might be more acceptable to the many people who hold the view that termination is preferable where it occurs earlier in pregnancy. Fifth, some women view drug-based regimens, particularly when used at home, as more natural or more compatible with their religious or ethical views than clinic-based surgical procedures.^1^ ^16^ Finally, though perhaps more controversially, where used without a prior pregnancy test, these treatments would allow the woman to become definitely *not* pregnant, without ever needing to confront as a certain fact that she had acted intentionally to end a known pregnancy. While this raises issues that I have no space here to explore, this blurring of the received boundary between contraception and abortion might be seen as an advantage that would render the technology attractive to some in a context where abortion is a far more stigmatised treatment.

Clearly, even if their safety and efficacy were to be established, these treatments would not be ideal for all women, in all instances. The regimens described above each have disadvantages compared with some other methods of fertility control. For example, they do not offer the protection from sexually transmitted diseases provided by barrier methods and they do nothing to challenge the embarrassment that results in the non-usage of such forms of contraceptive. Further, the fact that they would operate only early in pregnancy necessarily limits the number of cases in which they might be useful. Moreover, their development would need to overcome significant clinical obstacles. Raymond *et al* note:

Technically, development of a pharmaceutical regimen that reliably disrupts the pregnancy process after fertilisation, either before or after implantation or both, might be challenging. Progesterone receptor modulators such as mifepristone, given in adequate doses at certain times in the menstrual cycle, can inhibit endometrial implantation of a blastocyst. Mifepristone, particularly in combination with a prostaglandin, does have the well-established ability to terminate pregnancy when administered after implantation. However, its efficacy very early in gestation is unclear.^1^

Before such treatments might be made available, further research and robust clinical trials would be necessary to demonstrate their safety and efficacy in the various kinds of regimens discussed above. However, the advantages described above render these regimens worthy of such further investigation.

Yet, it seems unlikely that these treatments could be easily trialled or, if demonstrated to be safe and effective, made widely available for use in England. In each of the regimens noted above, the drugs are administered or provided with a clear intention to disrupt any pregnancy and, where they potentially operate after implantation, the elements of a criminal offence under the Offences Against the Person Act may be met. Further, as for menstrual regulation*,* in those cases where no positive test has established the existence of a pregnancy, it is unclear whether offering the drugs within the framework laid down in the Abortion Act might render their use lawful: as noted above, the Act offers protection in those cases where a pregnancy is ‘terminated’.^13^ Moreover, even if the Act's protection does extend to cases where a pregnancy is suspected but not proved, this would impose an extremely onerous regulatory framework for techniques that operate so early in pregnancy and one liable to undermine many of the advantages that they offer. Notably, the drugs could only be used following the authorisation of two medical practitioners, who had each taken a good faith view that the continuation of a given pregnancy (should one exist) would pose a greater risk to the woman's physical or mental health than would ending or preventing it; the administration of the drugs would need to be supervised by a doctor; and the drugs would need to be taken on the National Health Service or other approved premises.^10^ The convenience and simplicity of a regimen where women could have drugs in their bathroom cabinet, ready to be used when necessary, would be lost.

It might be suggested, of course, that this is all for the good: that terminating even a very early pregnancy should be treated as a morally serious matter and one that is rightly subject to strict control. After all, even if it can be convincingly established that such control is not needed for reasons of patient safety, it may nonetheless be justified through reference to the 1861 Act's purpose of preventing or condemning the intentional destruction of fetal life. Opinion on the broad, ongoing merits of this purpose in this context and, specifically, whether such onerous restrictions are justified so early in pregnancy will undoubtedly be divided. However, what might be agreed upon is that an issue of this significance to women's reproductive health should be decided on the basis of democratic debate, informed by current medical understandings of reproductive biology, careful reflection on the moral significance of implantation in the process of embryonic and fetal development and consideration of the regulatory implications of the ease with which drugs available in one country yet prohibited in another can now be obtained through online purchase.

No such informed consideration has preceded the adoption of implantation as the appropriate moral marker between the very different regulatory frameworks governing access to contraception and abortion. The abortion provisions of the 1861 Act were subject to no debate either within or outside of Parliament and, given the available medical technologies and more rudimentary understandings of reproductive biology in Victorian Britain, we can be confident that distinguishing between abortion and contraception was not in the minds of the architects of the 1861 Act.^17^ Moreover, the search for a good contemporary reason for according implantation such profound significance is equally elusive. I have been unable to find any survey canvassing public opinion on where the dividing line should fall between contraception and abortion, what factors should be relevant to this decision and what regulatory implications this should have. While the absence of such empirical data renders such a claim inevitably speculative, it seems at least possible that the contragestive regimens described above—tablets taken within weeks of intercourse, perhaps without a pregnancy ever being confirmed, at a stage when a large proportion of pregnancies will be naturally lost—might, for many, intuitively appear more appropriately regulated in the same way as contraceptives, with restrictions on access dictated only by concerns for patient safety.

Similarly, it should be noted that the philosophical literature dealing with abortion offers little support for the view that implantation is an important moral marker. While a voluminous literature offers careful, detailed, although greatly conflicting, justifications for the ethical significance of various points in embryonic and fetal development (including fertilisation, the emergence of the primitive streak, the acquisition of various cognitive abilities, the achievement of the capacity for independent life, birth, and so on),^18–20^ I have failed to find a single philosophical account that argues for the moral significance of implantation. Theological justifications are similarly elusive.

Two broader arguments in support of the significance of implantation should be noted, although each is ultimately unconvincing. First, it might be suggested that while implantation does not enjoy any particular ethical significance in its own right, it nonetheless offers a practically useful, conveniently timed marker for a necessary gear change between the appropriate regulation of contraception and abortion. This argument may well have had some force in a historical context where there was relatively clear water between known methods of disrupting a pregnancy that operate in a matter of days after intercourse, on the one hand, and techniques that are effective in interrupting an established pregnancy only after an interval of a significant number of weeks, on the other. For sure, menstrual regulation served to muddy this water; however, this technique never offered the potential advantages that come with the drug-based contragestive regimens described above and, as such, the fact that its use was legally blocked did not represent too serious a loss to the range of methods of fertility control available to English women. The contragestive techniques described above, however, operate precisely to remove the clear water between contraception and abortion and, given the potential advantages that they offer, existing legal hurdles to their development and use offer a serious challenge to any assumed ‘convenience’ of drawing a distinction on the basis of implantation.

Second, those who believe that the moral significance of the human conceptus lies in its potential to become a full human person might argue that implantation is important in representing a point of increased probability that a pregnancy will succeed, with one study finding that 22% of pregnancies are lost before the stage at which they might be clinically detected.^21^ But even accepting for the sake of argument that the point of transition between the very different regulatory frameworks described above is appropriately grounded in a calculation of the likelihood of a pregnancy succeeding to term (in itself, a controversial claim), this does not resolve the issue of where the boundary thus justified is best located. The same study found that 31% of pregnancies were lost after implantation,^21^ and 12 weeks is typically taken to be the point when a pregnancy is sufficiently certain for it to be ‘safe’ to communicate it to family and friends. The important point, again, is that any decision of whether and how such probabilities matter ethically should be the product of a modern, democratic debate, informed by current medical science.

## Conclusion

The regulatory cliff edge between ‘contraception’ and ‘abortion’ in English law results not from careful consideration but from historical accident. It has emerged as contraceptive use and availability has developed in line with evolving popular morality, with the language of the 1861 Act proving sufficiently flexible to accommodate the lawful use of modern contraceptive technologies.^9^ While attitudes to abortion, particularly in the early stages of gestation, have similarly become far more liberal since 1861, the limits to the elasticity of statutory language prevents any similar evolution that might allow for the regulation of early contragestive techniques to be governed merely by concerns for patient safety.

It is now over four decades since one commentator wrote of the 1861 Act:

The situation is that a law which even on its own proper ground was already antiquated is now being asked to bear an impossible yet increasing strain … Of course there must be a thorough overhaul of the crime of abortion, in all its aspects, before long.^22^

This call for reform has been frustrated by the reluctance of successive governments to address the issue of abortion, which in itself may reflect the dominance of vocal minorities in the public debate. Most recently, abortion law has been excluded from the Law Commission's otherwise wide-ranging consultation on the operation of the 1861 Act, a statute that it rightly recognises to be archaic, inconsistent and deeply flawed.^23^ The resulting legal stagnation leaves us with a criminal law framework for the voluntary interruption of pregnancy that is grounded in the moral mores and medical practices of earlier times. Moreover, while many countries treat ‘implantation’ as offering an important moral and legal boundary, this results in a regulatory cliff edge that is particularly sheer in English law, given the onerous criminal penalties contained in the 1861 Act and the tight medical control that the 1967 Act foresees for abortion to be deemed lawful. That archaic legislation, which has remained largely unconsidered for one and a half centuries, is drafted so as to block the development and use of safe, effective forms of fertility control that operate so soon after intercourse provides a compelling argument for a fundamental review of, at least, this aspect of its operation. Further, within any such review, given the reproductive health arguments in favour of facilitating access to safe, effective technologies that operate at early gestational ages, it seems reasonable to suggest that the onus should be on those who support the use of criminal sanction to provide good arguments to justify the deployment of this most punitive of state powers in this context and, specifically, to explain why it offers an appropriate response at such an early stage of pregnancy.^24^ ^25^

The fact that life exists as a seamless continuum means that the attempt to identify markers that allow us to make moral and legal distinctions between different stages of biological development is a fraught enterprise, with any purported bright lines liable to be criticised as misplaced or arbitrary. It does not follow, of course, that law can operate without such lines: we need to be able to determine when someone acquires the full human rights that accompany legal personhood and to distinguish between a living person and a dead body. While such lines will be inevitably (and appropriately) subject to contestation, it is important to ensure that the process by which they are drawn is capable of robust defence: that they are grounded in careful consideration, informed by clear moral reasoning and a solid medical evidence base. The modest claim defended in this paper is that the current basis for distinguishing between contraception and abortion falls woefully short of meeting this test. Rather, it is determined by a statutory phrase that is a product of a world, which ‘in matters sexual was almost unimaginably different from ours’ (para 332),^11^ having been passed by a Victorian Parliament within which women had no voice.^ii^ This is an indefensible basis for the regulation of health services that matter so intimately to modern women.

## Footnotes

i Munby J has since joined the Court of Appeal.

ii The first female Member of Parliament was elected in 1918, over 50 years after the passage of the 1861 Act. Women only gained the right to vote on equal terms to men 10 years later, under the Representation of the People (Equal Franchise) Act 1928.
